# Sour Ageusia in Two Individuals Implicates Ion Channels of the ASIC and PKD Families in Human Sour Taste Perception at the Anterior Tongue

**DOI:** 10.1371/journal.pone.0007347

**Published:** 2009-10-08

**Authors:** Taufiqul Huque, Beverly J. Cowart, Luba Dankulich-Nagrudny, Edmund A. Pribitkin, Douglas L. Bayley, Andrew I. Spielman, Roy S. Feldman, Scott A. Mackler, Joseph G. Brand

**Affiliations:** 1 Monell Chemical Senses Center, Philadelphia, Pennsylvania, United States of America; 2 The Monell-Jefferson Taste and Smell Clinic, Department of Otolaryngology-Head & Neck Surgery, Thomas Jefferson University, Philadelphia, Pennsylvania, United States of America; 3 New York University College of Dentistry, New York, New York, United States of America; 4 Veterans' Affairs Medical Center, Philadelphia, Pennsylvania, United States of America; 5 School of Dental Medicine, University of Pennsylvania, Philadelphia, Pennsylvania, United States of America; 6 Department of Medicine, University of Pennsylvania, Philadelphia, Pennsylvania, United States of America; Duke University, United States of America

## Abstract

**Background:**

The perception of sour taste in humans is incompletely understood at the receptor cell level. We report here on two patients with an acquired sour ageusia. Each patient was unresponsive to sour stimuli, but both showed normal responses to bitter, sweet, and salty stimuli.

**Methods and Findings:**

Lingual fungiform papillae, containing taste cells, were obtained by biopsy from the two patients, and from three sour-normal individuals, and analyzed by RT-PCR. The following transcripts were undetectable in the patients, even after 50 cycles of amplification, but readily detectable in the sour-normal subjects: acid sensing ion channels (ASICs) 1a, 1β, 2a, 2b, and 3; and polycystic kidney disease (PKD) channels PKD1L3 and PKD2L1. Patients and sour-normals expressed the taste-related phospholipase C-β2, the δ-subunit of epithelial sodium channel (ENaC) and the bitter receptor T2R14, as well as β-actin. Genomic analysis of one patient, using buccal tissue, did not show absence of the genes for ASIC1a and PKD2L1. Immunohistochemistry of fungiform papillae from sour-normal subjects revealed labeling of taste bud cells by antibodies to ASICs 1a and 1β, PKD2L1, phospholipase C-β2, and δ-ENaC. An antibody to PKD1L3 labeled tissue outside taste bud cells.

**Conclusions:**

These data suggest a role for ASICs and PKDs in human sour perception. This is the first report of sour ageusia in humans, and the very existence of such individuals (“natural knockouts”) suggests a cell lineage for sour that is independent of the other taste modalities.

## Introduction

The human organ of taste is the tongue. The dorsal surface of the human tongue projects four types of papillae. The cornified filiform papillae which give the tongue its roughness are invested with sensory fibers, but contain no taste buds. Taste buds are located on the remaining three types of papillae: on the fungiform papillae at the tip and on the anterior two thirds of the tongue, each papilla expressing zero to about 12 or more taste buds; the foliate region (not strictly a papilla) at the lateral edges of the tongue about three-quarters of the way toward the posterior, each one showing 50 to 125 buds; and the 6 to 8 circumvallate (or, simply, the vallate) papillae on the posterior dorsal surface, each possessing from 100 to 200 buds. Extra-lingual taste buds are located in the area of the soft palate and pharynx. As tongue topography is species specific (for example, the bovine has no foliate region and the cat has a set of “clavate papillae” instead of foliates), generalizing this description to other species should be made with caution [1].

Each taste bud is a collection of ∼50–75 cells which can be categorized into various types, each type having a specialized role to play in transduction and development. Originally, the cells of the bud were typed by their morphology, but recent molecular analyses are subdividing the simple classification into types defined by their apparent function [2].

The taste bud is equipped with cells possessing receptor and transduction elements such that the five primary tastes in human – sweet, salty, sour, bitter and umami (savory) – can be discriminated and manipulated independently of each other. Much progress has been made in recent years in decoding the molecular mechanisms underlying each of these taste qualities [3]. A G protein coupled receptor system transduces the qualities of sweet, bitter and umami, while salty and sour taste are thought to be mediated by ion channels. The nature of these channels, and their relative importance for the ultimate experience of the sensations of saltiness or sourness, remain elusive, especially in the human.

Sourness – the subject of this work - is associated with acids. Since all acids release protons in solution, sour taste would appear to be a matter of proton sensing, i.e., detecting the concentration of hydrogen ions (pH). However, such is not the case. Rather sourness is correlated much better with titratable acidity, implying that the receptor(s) for sour taste is a proton counter [4], [5].

In spite of the efforts of many laboratories, no consensus has been reached defining the receptor mechanism for sour taste. There are several reasons for this including the possibility of species specific transduction processes and, even within a given species, processes specific to a particular region of the tongue. There are at least a dozen such proposed mechanisms, all of which are supported by experimental evidence [6], [7]. Given these species specificities, it seems possible that humans may use molecular processes that are unique to old world primates. To explore the likely sour taste mechanisms, it would be helpful to have antagonists of sour taste in humans or, alternatively, humans who have lost their sense of sour taste.

Regarding this latter need, we identified two patients at the Monell-Jefferson Taste and Smell Clinic (MJTSC) who were found to be sour-ageusic. Psychophysical taste tests confirmed their inability to identify sour stimuli while finding a normal ability to identify and taste the other modalities.

Assuming the defect in sour taste of these two patients to be peripheral, i.e., at the level of the taste bud, we saw their psychophysical documentation as a firm anchor from which to search for suspected molecular elements of sour taste. We chose as molecular targets those ion channels shown not only to be affected by sour stimuli, but also responsive to the sourness of the stimuli rather than only to their pH.

Since controversy currently brews over three of these channel mechanisms, we saw the search for transcripts of these molecular targets in the sour-ageusics as an opportunity to help resolve this debate:

1. One mechanism gives a central role in sour taste transduction to the acid sensing ion channels (ASICs). These comprise a family of proton-gated ion channels related to the superfamily of degenerins/epithelial sodium channels [8]–[12]. Several ASICs have been localized to taste tissue and it is notable that their expression is both species specific and often restricted to only one region of the tongue [13]–[15].
2. Another mechanism assumes that two members of the polycystic kidney disease (PKD) family of channels, namely, PKD1L3 and PKD2L1, are involved at least in part [16]–[18]. These are proton activated channels of the TRP (transient receptor potential) superfamily and are co-expressed in taste cells of the mouse vallate and the foliate, but with only PKD2L1 reportedly detectable in rodent fungiform taste cells. Genetic ablation of PKD2L1-expressing cells resulted in mice unresponsive to sour stimuli [18].
3. A third mechanism assumes the receptor is the heterotrimeric epithelial sodium channels (ENaC), which have been implicated in the sourness mechanism in hamsters [19]. These channels are found in all epithelia, including the taste bud, where in the human, the more abundant alpha subunit is replaced by the delta subunit, which can be activated by protons [20]. It is widely assumed that ENaCs are the major channels mediating salty taste. Their absence in the taste tissue of these sour-ageusic patients would call this assumption into question.

Protons, being chemically very active, affect virtually every class of ion channel [21] as well as a number of metabolic pathways. Using cells or tissues from experimental animals, it is difficult to differentiate the specific impact of protons on sour transduction from their more general effects. With human subjects, however, we have a model system wherein we can ask a psychophysical question and receive a molecular answer.

In this study, we used RT-PCR to search for evidence of expression of the ASIC, PKD and ENaC families in the anterior fungiform (taste) papillae, in both the sour-ageusics and in three normal subjects. Major differences were found in the level of expression of the ASIC and PKD channels between the two groups, yet both groups expressed comparable levels of δ-ENaC and other taste bud specific genes.

If all of the ASICs and PKD channels play roles in peripheral detection of sourness, then, given the dynamic range in pH over which sourness can be detected, it may be plausible that sour transduction involves multiple proton-gated heteromeric channels. This point has been explicitly emphasized in studies of acid sensing in the central nervous system [22] and in the gastrointestinal tract [23].

We use the word “sourness” as a reflection of molecular mechanisms that induce that sensation, but strictly speaking, “sourness” is a sensation that exists only in the brain of an observer. Thus, receptors cannot monitor “sourness” but rather some chemical condition that is a direct correlate of sourness – e.g. total titratable acidity. But since the focus of our work is taste transduction, we will use the term “sourness” to refer to the collective processes that induce that sensation.

Preliminary accounts of some of these studies have been presented at professional meetings and symposia [15], [24], [25].

## Results and Discussion

### Documentation of the sour-ageusic diagnosis of two patients seen at the Monell/Jefferson Taste and Smell Clinic (MJTSC)

In this study we report psychophysical results demonstrating a sour ageusia in two patients seen at the MJTSC. To the best of our knowledge, this is the first report of such a syndrome in humans. These two patients act as “natural knockouts”, analogous with “knockout mice”, giving us the opportunity to ask the following related questions: (1) Is expression of the genes for putative sour receptors undetectable in fungiform papillae from the two sour-ageusics?; and (2) Is expression of these same genes readily detectable in control subjects with normal sour perception? Further, we also sought to determine if genes associated with modalities other than sour were expressed in the fungiform papillae of both patients and control subjects.

Details of the psychophysical testing procedures and response criteria for abnormality used by the MJTSC have been described previously [26]. Of the more than 1,500 patients seen by the MJTSC in the past 25 years, only 2 have been documented as sour-ageusic, without, at the same time, showing evidence of a severe, generalized taste deficit.

Patient 8689, an 83 year old African-American male, presented to the clinic in 2005 with the complaint of a complete loss of smell and diminished taste. He attributed his chemosensory loss to a difficult recovery period following cardiac surgery five years previously. In addition to his chemosensory problems, he reported some hearing loss and problems with vision shortly after the surgery. But unlike the chemosensory disorders, these auditory and visual problems resolved themselves within a year after surgery. He was diabetic, using insulin, along with the following medications: atenolol, amlodipine, furosemide, low dose aspirin, simvastatin, warfarin and amitriptyline. The patient was a former smoker but at the time of evaluation he had not smoked for many years.

Patient 8689 was found to be anosmic. His “taste” complaint appeared to largely reflect retronasal olfactory flavor loss. However, in a routine taste screening measure, he failed to respond consistently to suprathreshold citric acid (sour) stimuli (1.8–18 mM), although his responses (quality identification and intensity ratings) to suprathreshold sucrose (sweet), NaCl (salty) and quinine sulfate (bitter) were all within normal limits.

A second, more focused, forced-choice threshold assessment confirmed that Patient 8689 was unable to reliably discriminate citric acid solutions up to 18 mM from water, although he occasionally reported perceiving a tingling sensation at high concentrations. His threshold for NaCl (6.5 mM) was within normal limits. Thresholds for other taste qualities were not assessed because of subject fatigue.

A second individual, Patient 8716, a 62 year old white female, presented to the Clinic in 2006 with a complaint of markedly diminished taste sensitivity. She traced this taste problem, as well as a now largely resolved loss of smell, to an upper respiratory infection in June of 2000. At the time of evaluation in our clinic, she reported taking furosemide, triamterene, levothyroxine, alendronic acid and a potassium supplement. She was a former smoker but at the time of evaluation she had not smoked for many years.

For Patient 8716, psychophysical testing revealed an impairment in retronasal olfactory flavor perception, which probably contributed to her “taste” complaint. In addition, on our suprathreshold taste screening measure, she, like Patient 8689, failed to correctly identify or respond to the sour stimuli, although she reported tasting, and was able to identify, the sweet, salty and bitter stimuli. Her detection threshold for citric acid was markedly elevated at 7.5 mM. In our database, a citric acid threshold >0.2 mM is considered abnormally high for women. She showed moderately diminished salt sensitivity as well (detection threshold concentration of 56 mM, with thresholds >10 mM being considered abnormal for women). Her detection thresholds for the sweet and bitter stimuli were within normal limits. Interestingly, even though the patient correctly identified sweet, salty and bitter stimuli, and the thresholds of each were normal or slightly high normal, her ratings of the intensity of the three stimuli were low and not concentration dependent. Her subjective reporting of a loss of taste in general may be related to the low and inconsistent intensity ratings for these three pure stimuli. This possible separation of taste quality from intensity should be investigated further.

It will be noted that the threshold for salt is obviously higher than that for citric acid, and one may wonder why the salt threshold is considered a moderate loss and the sour threshold profound. The explanation is that although salt and citric acid are gustatory stimuli, both are also oral trigeminal stimulants at higher concentrations and should be detectable through that sense at concentrations >400 mM NaCl and >1 mM citric acid [26]. Therefore, the citric acid threshold for Subject 8716 is probably a trigeminal, and not a taste, threshold, whereas the threshold for salt, despite being elevated, is nonetheless a taste threshold that is well below that required for oral trigeminal stimulation.

In summary, the diagnosis from the evaluation of these two patients was that both were sour-ageusic.

### Etiology of the sour ageusia

Both patients were elderly. The relationship between aging and taste is well appreciated, even if it is somewhat idiosyncratic [27]. While normal aging might entail a certain susceptibility to general taste impairment, at times the impairment can be quality specific [28]. The two patients described here gave intensity responses to various taste stimuli that were generally within normal limits, with the exception of sour taste, where they exhibited complete ageusia. Because of the apparently normal responses to all stimuli except sour, we believe the role of aging in the development of sour ageusia in these two patients to be small.

The compromised general health of these patients is reflected by the number of medications being taken. The impact of medications on taste has been the subject of several studies and a number of case reports [27], [28]. The Physicians' Desk Reference (PDR) reports taste loss or distortion as an adverse reaction [29]. According to the PDR, none of the medications that Patient 8689 reported taking at the time of appearance at the clinic can be associated with alterations in taste. For alendronic acid, which Patient 8716 was taking, the PDR reports one study showing a 0.5% incidence of a “taste perversion” with this drug. (We noted that the placebo group reported a 1% incidence of an unspecified taste perversion.) The only medication both patients had in common, furosemide, is not, according to the PDR, associated with taste problems.

On the other hand, there are claims elsewhere of possible taste alteration while taking aspirin, simvastatin, amitriptyline, and triamterene [27]. Simvastatin and amitriptyline function as substrates of P-glycoprotein, which is an important component of the blood-brain barrier which transports drugs back into the vascular space [30], [31]. The combined effect of these drugs may have resulted in their plasma concentrations being elevated to supratherapeutic levels. Amitriptyline is known to inhibit the growth of olfactory and cerebral neurons *in vitro* at doses similar to the plasma concentrations known to be therapeutic in humans [32]. This could explain the anosmia of Patient 8689, and it may also be the underlying cause, at least in part, for his sour ageusia.

In addition, the impact of critical illnesses can have major deleterious effects on sensory perception, as has been emphasized [33]. In our study, this would have been an important factor at least for Patient 8689, for whom the medically significant trauma was cardiac surgery, after which he reported diminished visual capacity along with chemosensory problems.

Another possible underlying cause of the sour ageusia might be inflammation. It has been shown recently that inflammation resulting from bacterial and viral infection activates interferon signaling pathways in taste bud cells, affecting their function in taste transduction. Moreover, the apoptosis that is also induced may cause abnormal cell turnover and thereby skew the representation of different taste bud cell types, leading to the development of taste disorders [34]. This mechanism may be relevant as an explanation for the sour ageusia of Patient 8716, with her history of an upper respiratory infection.

It is of interest to note the recent publication of a case report describing an individual with myasthenia gravis who had a sweet-specific ageusia [35].

In summary, we have documented a specific sour ageusia in two patients seen at the MJTSC. Regardless of the etiology of the sour aguesia, comparison of likely molecular components of sour transduction between these sour-ageusic patients and normal volunteers may, by difference, provide clues to the mechanisms of sour taste transduction. As such, these patients provide us with a window into the molecular mechanisms of sour taste transduction. Whatever the ultimate cause of the sour ageusia, it was powerful enough and precise enough to completely yet very specifically eliminate sour taste. This observation in itself predicts the existence of a direct lineage of developing cells whose programmed existence is for the purpose of maintaining a sour taste modality.

### RT-PCR of taste-related transcripts in fungiform papillae of the two sour-ageusic patients and normal controls

Table 1 lists the transcripts and genes that were probed for in this study, along with the primers used to detect their expression by RT-PCR. Based on prior literature we labeled as probable sour related genes the following: ASICs 1a, 1β, 2a, 2b and 3; PKD1L3 and PKD2L1; and δ-ENaC. In 2002 we reported that the delta subunit is preferentially expressed and abundant in the human taste bud [36]. We also considered it likely that δ-ENaC was involved in salty taste, predicting its expression in both patients and controls in the current study. Other genes sought included the ubiquitous β-actin; the taste bud-specific phospholipase C-β2 (PLC-β2), which is involved in the transduction pathways of both bitter and sweet receptors [37]; and a bitter taste receptor activated by multiple ligands, T2R14 [38].

**Table 1:** List of primers used in this study.

Target Transcript Or *Gene*	GenBank Accession Number	Primer Name	Orientation	Sequence 5′→3′	Product Size (bp)
ASIC1a	U78181	BNACF11	Forward	CAACAAGGATGGAACTGAAGGCCGA	
		BNACR12	Reverse	ATCTAGGCCTTTGGTTCAGCGG	1627
ASIC1a	U78181	HASIC3	Forward	GTACTGCGTGTGTGAAATGCC	
		HASIC4	Reverse	TGTTGGCAGCGTATGTCATC	461
*ASIC1*	U78181	HASIC3	Forward	GTACTGCGTGTGTGAAATGCC	
		HASIC4	Reverse	TGTTGGCAGCGTATGTCATC	1535
*ASIC1*	U78181	ASICMCF1	Forward	TGGCCCACATCTTCTCCTAC	
		ASICMCR1	Reverse	CATCTGCCATCTGTGTGTCT	936
ASIC1β	AJ006519	ASICβF1	Forward	ATGGAGGCAGGGTCGGAGTT	
		ASICβR1	Reverse	GGCCCCACAGTAGGAACAA	490
ASIC2a	U57352	HBNAC1AF	Forward	ACAGGAGCAGAGGCTCACAT	
		HBNAC1DR	Reverse	TGAACAATCCCATCTGACCA	500
ASIC2b	Y14635	RASIC2BF2	Forward	CACTAAATTGCACGGGCTG	
		RASIC2BR2	Reverse	GCATATCCTCCAGCTGGTG	467
ASIC3	AF057711	HDRASICF2	Forward	TTCACCACGATCTTCACCCG	
		HDRASICR2	Reverse	ACGTCGCCTGGCATGTACAC	491
β-actin	NM_001101	HBACTINF1	Forward	ATGGATGATGATATCGCCGCGC	
		HBACTINR1	Reverse	CTAGAAGCATTTGCGGTGGAC	1128
β-actin	NM_001101	HBACTINF2	Forward	CGTGACATTAAGGAGAAGCT	
		HBACTINR2	Reverse	CATACTCCTGCTTGCTGATC	460
β-actin	NG_003162	ACTINX4F. ACTINX5R	Forward, Reverse	TCATGTTTGAGACCTTCAA, GTCTTTGCGGATGTCCACG	506
PKD1L3	AY164485	HPKD1F1	Forward	GAACTCTGCTGCGACTCACC	
		HPKD1R1	Reverse	TGTCACTGCCCACTGCTGTCGT	530
PKD2L1	NM_016112	HPKD2F1	Forward	ACACTGAGATTGAGAAACTAGGCCG	
		HPKD2R1	Reverse	GCCTCACACTTAACTCCTCTGC	410
*PKD2L1*	NM_016112	HPKD2F1	Forward	ACACTGAGATTGAGAAACTAGGCCG	
		HPKD2R1	Reverse	GCCTCACACTTAACTCCTCTGC	2123
PLCβ2	NM_004573	PLCBF1	Forward	AGGAGCAGTACGAGTGCGTT	
		PLCBR1	Reverse	CTTCACCTCTGCCTCCAGAC	431
T2R14	AF227138	BTR14F1	Forward	ATGGGTGGTGTCATAAAGAG	
		BTR14R1	Reverse	TCAAGATGATTCTCTAAATTCT	954
δ-ENaC	U38254	HENACDF1	Forward	ATGGCTGAGCACCGAAGCATGGAC	
		HENACDR3	Reverse	GAGGTTGACGTTGTACAGGGA	501
δ-ENaC	U38254	HENACDF1	Forward	ATGGCTGAGCACCGAAGCATGGAC	
		HENACDR1	Reverse	GGTGTCCAGAGTCTCAAGGGG	1917
α-ENaC	L29007	HENACAF1	Forward	ATGGAGGGGAACAAGCTGGAGGAG	
		HENACAR1	Reverse	GGAGCATCTGCCTTGGTGTGAG	2048

Three control subjects (identified here as Subjects 45, 65 and 49: see Methods for age and ethnicity) participated in the biopsy procedure wherein from 6 to 8 fungiform papillae were removed from the tongue of each volunteer. Likewise, Patient 8689 and Patient 8716 consented to donate fungiform papillae by surgical biopsy.

**Figure 1:**
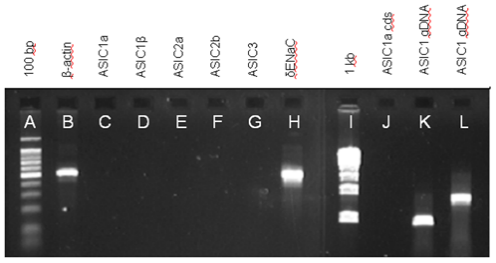
RT-PCR and genomic analysis of acid sensing ion channel (ASIC) gene expression for sour-ageusic Patient 8689. The patient expresses housekeeping and taste-related genes but ASIC transcripts are undetectable even after 50 cycles of amplification, and for ASICs 1a and 1β this is not the result of a loss of the *ASIC1* gene. Lane identifications: (A) 100 bp DNA marker with the brightest band being 500 bp; (B) Transcript of β-actin; (C–G) Transcripts of, respectively, ASICs 1a, 1β, 2a, 2b and 3; (H) Transcript of δ-ENaC; (I) 1 kb DNA marker; (J) ASIC1a, full coding sequence; (K) genomic DNA amplified with primer pair ASICMCF1 and ASICMCR1 (see Table 1); and (L) genomic DNA amplified with primer pair HASIC3 and HASIC4 (see Table 1). RT-PCR was performed with cDNA from fungiform papillae (B–H and J), but only the data for the tubes containing reverse transcriptase are presented. No products were detected in the tubes lacking reverse transcriptase. Genomic analysis (K, L) was performed using buccal tissue. Individual amplification reactions were performed for each named molecular target and the data are shown in collated form. Identities of all amplification products were confirmed by sequencing.

RT-PCR of cDNA from the fungiform papillae of Patient 8689 showed that a 506-bp transcript for β-actin was clearly expressed (Figure 1, Lane B). However, the full coding sequence of ASIC1a, amplified with primers BNACF11 and BNACR12, was not detectable (Figure 1, Lane J), even after 50 cycles (virtually all transcripts can be detected after 25–40 cycles in standard amplification protocols). Detection of a shorter fragment of ASIC1a (461 bp) with primers HASIC3 and HASIC4, also yielded negative results even after 50 cycles (Figure 1, Lane C). Finding no transcripts for ASIC1a we used RT-PCR to search for transcripts for ASIC1β, ASIC2a, ASIC2b, and ASIC3. These also were not detected (Figure 1, Lanes D–G), using the primers listed in Table 1. But a 501-bp fragment of the transcript for δ-ENaC was clearly expressed, using primers HDENACF1 and HDENACR3 (Figure 1, Lane H).

**Figure 2:**
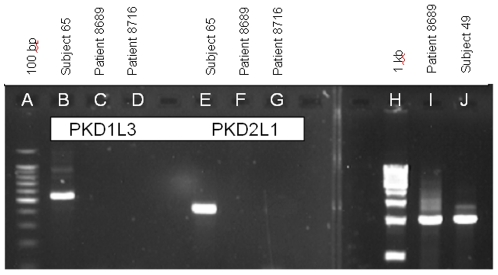
RT-PCR and genomic analysis of polycystic kidney disease (PKD) gene expression in sour-ageusic patients and sour-normal subjects. Sour-ageusic patients do not express detectable transcripts for either PKD1L3 or PKD2L1, even after 50 cycles of amplification, and for PKD2L1 this is not the result of a loss of the *PKD2L1* gene. Sour-normal subjects express both transcripts. Lane identifications: (A) 100 bp DNA marker with the brightest band being 500 bp; (B–D) Transcript for PKD1L3 in, respectively, sour-normal Subject 65, sour-ageusic Patient 8689 and sour-ageusic Patient 8716; (E–G) Transcript for PKD2L1 in, respectively, sour-normal subject 65, sour-ageusic Patient 8689 and sour-ageusic Patient 8716; (H) 1 kb DNA marker; (I) genomic DNA of Patient 8689 amplified with primer pair HPKD2F1 and HPKD2R1 (see Table 1); (J) RT-PCR for full coding sequence of PKD2L1 of sour-normal Subject 49. RT-PCR was performed with cDNA from fungiform papillae (B–G and J), but only the data for the tubes containing reverse transcriptase are presented. No products were detected in the tubes lacking reverse transcriptase. Genomic analysis (I) was performed using buccal tissue from Patient 8689. Patient 8716 did not provide buccal tissue. Individual amplification reactions were performed for each named molecular target and the data are shown in collated form. Identities of all amplification products were confirmed by sequencing.

Also not detected in the fungiform cDNA of Patient 8689 were the two channels, PKD1L3 and PKD2L1, even after 50 cycles of amplification (Figure 2, Lanes C and F). These transcripts were clearly expressed in the fungiform papillae of sour-normal Subject 65 (Figure 2, Lanes B and E).

Patient 8689's apparent complete lack of transcripts for the ASICs and PKDs led us to consider the very remote possibility that the genes for these proteins were compromised. The patient consented to a cheek swab for genomic DNA analysis. The initial analysis of the *ASIC1* gene showed that at least the 5′ end was present, when using the primers ASICMCF1, located in Exon 2 and beginning at base 89 of the coding sequence, and ASICMCR1, located in Exon 3 and ending at base 394 of the coding sequence. The intervening intron is of size 630 bp, so that amplification of genomic DNA would be expected to yield a product of size 936 bp, which was indeed seen (Figure 1, Lane K). Subcloning and sequencing of this product confirmed its identity (100%) as *ASIC1*.

An additional test for the presence of the *ASIC1* gene was employed, this time using primers spanning the 3′ end. To this end we used the primer pair HASIC3, located in Exon 8 and starting at base 1076 of the coding sequence, and HASIC4, located in Exon 12 and ending at base 1536 of the coding sequence. The intervening introns 8, 9, 10 and 11 are of sizes 442, 374, 78 and 180 bp respectively. Amplification of genomic DNA with HASIC3 and HASIC4 would therefore be expected to yield a product of size 1535 bp. As shown, (Figure 1, Lane L), amplification of the genomic DNA of Patient 8689 with HASIC3 and HASIC4 did yield a product of the expected size. Again, subcloning and sequencing of this product confirmed its identity as *ASIC1*. Therefore, an apparently normal *ASIC1* was present in the patient's genome but its product was not detectable in the peripheral tissue of the taste papillae.

Similarly, for the putative sour receptor gene *PKD2L1*, amplification of the patient's genomic DNA was performed using the same primer pair used for RT-PCR: HPKD2F1, located in Exon 13 and starting at base 2018 of the coding sequence, and HPKD2R1, located in Exon 16 and ending at base 2427 of the coding sequence. The intervening introns 13, 14 and 15 are of sizes 262, 958 and 493 bp respectively, so that amplification of genomic DNA would be expected to yield a product of size 2123 bp. A product of this size was indeed obtained (Figure 2, Lane I). Subcloning and sequencing of this product confirmed its identity as *PKD2L1*. Therefore, *PKD2L1* was present in the patient's genome but not expressed in the peripheral tissue of the taste papillae.

In summary, for the sour-ageusic Patient 8689, we could detect no transcripts for ASIC1a, ASIC 1β, ASIC2a, ASIC2b, ASIC3, PKD1L3 and PKD2L1. However, β-actin was readily detectable, but this is not surprising in view of its abundance in virtually all cell types. Of greater relevance is our ability to detect a transcript for δ-ENaC. This transcript is far less abundant than β-actin, and the corresponding protein is also expressed in many taste cells but not in surrounding epithelium (please see below in the section entitled “Immunohistochemistry for putative taste-related proteins”). Given this taste cell specificity and the fact that both patients and normal subjects expressed δ-ENaC in their fungiform papillae, we conclude that even if it has a role in sour taste, it still plays an additional role in the taste bud, most likely as a receptor for saltiness.

These data on δ-ENaC clearly show that it can be considered a control gene for our studies, especially since the encoded ion channel is activated by protons (20), and it also proves that the tissue obtained from Patient 8689 contained taste cells. It also indicates that our inability to detect transcripts for ASICs and PKDs in this patient was not due to any problems with RNA extraction or with the subsequent cDNA amplification. We had insufficient cDNA from Patient 8689 to search for other taste related transcripts, and could not obtain additional material. With the papillae cDNA of the other individual, Patient 8716, we sought first to confirm the observations from Patient 8689, and then to search for taste-related transcripts.

**Figure 3:**
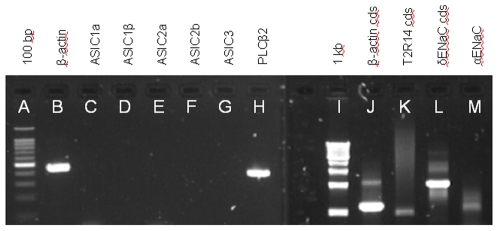
RT-PCR analysis of gene expression for sour-ageusic Patient 8716. The patient expresses transcripts for housekeeping genes and those involved in sweet, bitter and salty taste, but ASIC transcripts are undetectable even after 50 cycles of amplification. Lane identifications: (A) 100 bp DNA marker with the brightest band being 500 bp; (B) Transcript for 460-bp fragment of β-actin; (C–G) Transcripts for, respectively, ASICs 1a, 1β, 2a, 2b and 3; (H) Transcript for PLC-β2, which is involved in the transduction pathways of sweet and bitter; (I) 1 kb DNA marker; (J) full coding sequence of β-actin; (K) full coding sequence of bitter receptor T2R14; (L) full coding sequence of δ-ENaC; and (M) partial coding sequence of α-ENaC. RT-PCR was performed with cDNA from fungiform papillae but only the data for the tubes containing reverse transcriptase are presented. No products were detected in the tubes lacking reverse transcriptase. Individual amplification reactions were performed for each named molecular target and the data are shown in collated form. Identities of all amplification products were confirmed by sequencing.

The cDNA from the fungiform papillae of Patient 8716 was analyzed by RT-PCR using primers as above. The results resembled those of Patient 8689. No transcripts for any of the five major ASICs were detected (Figure 3, lanes C–G). Yet, expression of the housekeeping gene β-actin was unambiguous, with amplification of both a 460-bp fragment (Figure 3, Lane B) as well as the entire coding sequence (Figure 3, Lane J). Subcloning and sequencing of both actin products confirmed their identities (>99% similarity with the reference sequence listed in Table 1). It is relevant to mention here that for all the transcripts probed in this study, 3–6 independent clones were analyzed and there were no inconsistencies among clones in the sequence data obtained for any transcript.

To confirm that the cDNA from Patient 8716 included taste cell-related transcripts, we performed RT-PCR for expression of three taste-related genes. We readily detected transcripts for phospholipase C-β2, (Figure 3, Lane H), and for the bitter taste receptor T2R14 (Figure 3, Lane K). To confirm the identity of the transcript for T2R14, we sequenced the entire open reading frame, finding 100% deduced amino acid identity with the reference sequence listed in Table 1. PLC-β2, which is expressed specifically in the taste bud (see below in the section entitled “Immunohistochemistry for putative taste-related proteins”), participates in the transduction pathways of both bitter and sweet receptors [37], while the bitter taste receptor T2R14, which is activated by multiple bitter compounds [38] is, in our experience, relatively abundant in human fungiform papillae.

Also detected in the fungiform cDNA of Patient 8716 was the transcript for the entire coding sequence of the taste bud-associated δ-ENaC subunit (Figure 3, Lane L). Again, the presence of this control transcript indicates that the tissue obtained from this patient contained taste cells and that there were no methodological problems with RNA extraction. To confirm the identity of the subunit, the product was sequenced. The deduced amino acid sequence of the δ-ENaC of Patient 8716 showed 99% identity with the reference sequence listed in Table 1. We were unable to amplify a complete coding sequence transcript for the alpha subunit of human ENaC, although fragments were sometimes detected (Figure 3, Lane M). This fragment had an apparent size only one-half of the expected size. Sequencing showed that the predicted protein had 333 amino acids instead of 669, with a deletion extending from amino acid 48 to amino acid 383. Amino acids 1–47 and 384–669 of this mutant were 100% identical with those of the reference sequence listed in Table 1. The molecular mechanism(s) responsible for producing this deletion were not investigated further.

Patient 8716 also did not express transcripts for PKD1L3 and PKD2L1 (Figure 2, Lanes D and G). Patient 8716 did not provide a sample of buccal tissue for genomic DNA analysis.

**Figure 4:**
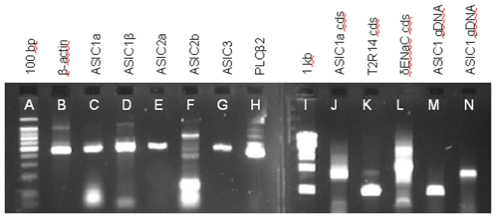
RT-PCR and genomic analysis of gene expression in sour-normal subjects. Transcripts for all ASICs tested are clearly detectable, as also are those for housekeeping and taste-related genes. Lane identifications: (A) 100 bp DNA marker, with the brightest band being 500 bp; (B) Transcript for 460-bp fragment of β-actin in a pooled sample of fungiform papillae from Subjects 45 and 49; (C–G) Transcripts for, respectively, ASICs 1a, 1β, 2a, 2b (weakly-staining band at 467 bp in Lane 6), and ASIC3 in a pooled sample of fungiform papillae from Subjects 45 and 49; (H) Transcript for PLC-β2 in Subject 49; (I) 1 kb DNA marker; (J) full coding sequence of ASIC1a of Subject 49; (K) full coding sequence of bitter receptor T2R14 of Subject 49; (L) full coding sequence of δ-ENaC of Subject 45; (M) genomic DNA of Subject 65 amplified for *ASIC1* gene with primer pair ASICMCF1 and ASICMCR1 (see Table 1); and (N) genomic DNA of Subject 65 amplified for *ASIC1* gene with primer pair HASIC3 and HASIC4 (see Table 1). RT-PCR (B–H and J–L) was performed with cDNA from fungiform papillae but only the data for the tubes containing reverse transcriptase are presented. No products were detected in the tubes lacking reverse transcriptase. Genomic analysis (M, N) was performed using buccal tissue. Individual amplification reactions were performed for each named molecular target and the data are shown in collated form. Identities of all amplification products were confirmed by sequencing. All three sour normal subjects expressed all the named transcripts that were probed for, but to avoid repetition of the same pattern of data only selected transcripts are shown.

In contrast with the cDNA of the two sour-ageusics above, cDNA from fungiform papillae of the sour-normal subjects contained transcripts for ASIC 1a, ASIC 1β, ASIC 2a, ASIC 2b, and ASIC 3 (Figure 4, Lanes C–G respectively, representing fragments of the coding sequences; and Lane J representing the entire coding sequence of the ASIC1a of Subject 49). Also clearly detected were transcripts for PKD1L3 and PKD2L1 (Figure 2, Lanes B and E representing fragments of the coding sequences for Subject 65; and Figure 2, Lane J representing the entire coding sequence of the PKD2L1 of Subject 49). The identity of each of these seven transcripts was confirmed by subcloning and sequencing. All three sour normal subjects expressed all the named transcripts that were probed for, but to avoid repetition of the same pattern of data only selected transcripts are shown. Assuming our supposition, stated previously, that the sour-ageusics would likely lack transcripts for proteins specifically involved in sour taste transduction, then the fact that the sour-normal subjects expressed all of these seven genes suggests the involvement of all of them somewhere along the sour taste transduction pathway.

In addition to these likely sour taste–related transcripts, the fungiform cDNA from sour-normal subjects also contained transcripts for β-actin, (Figure 4, Lane B, representing the pooled papillae of Subjects 45 and 49); PLC-β2 (Lane H, representing Subject 49); T2R14 (Lane K, representing the entire coding sequence of Subject 49); and δ-ENaC (Lane L, representing the entire coding sequence of Subject 45). The sour-normal subjects also expressed the *ASIC1* gene and as an example, the results from Subject 65 are shown (Figure 4, Lanes M and N, representing amplification of the 936 bp and 1535 bp products respectively).

### Immunohistochemistry for putative taste-related proteins

To confirm that the fungiform taste papillae actually express the proteins from transcripts of ASICs, δ-ENaC, PLC-β2, PKD1L3 and PKD2L1, we performed immunohistochemistry on taste bud-containing human fungiform papillae biopsied from individuals with normal taste perception. The biopsied papillae from the sour ageusic patients were used for RT-PCR analysis. Following that first biopsy, neither patient was available for another donation of fungiform papillae.

**Figure 5:**
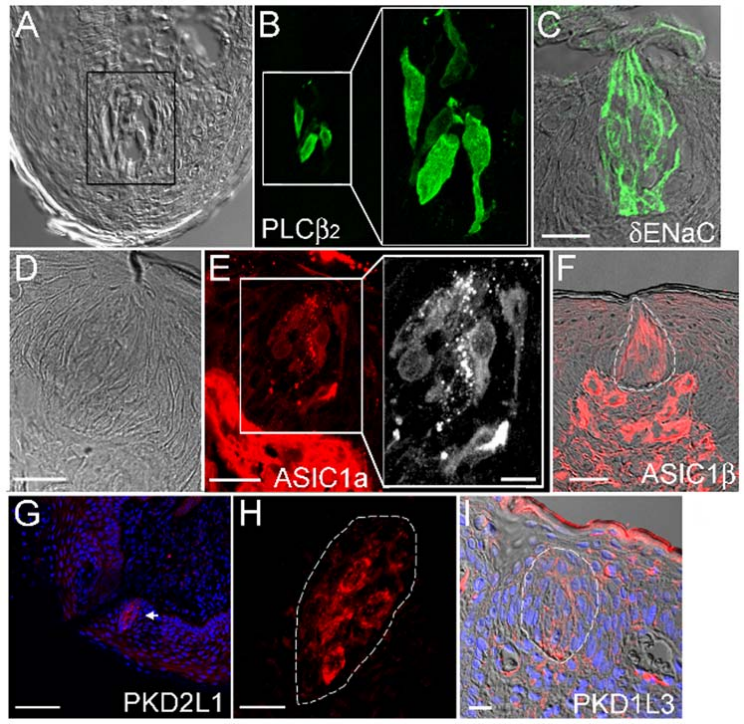
Immunohistochemical staining of the protein products of taste-related genes expressed in the fungiform papillae of sour-normal subjects. The gray image of Panel A is a fungiform section showing taste buds under Nomarski optics. The image in Panel B is of the same section as A, but showing the section immunostained with antibodies to PLC-β2. Panel C displays immunostaining with antibody to δ-ENaC, overlaid on a Nomarski picture of the same tissue section. The human taste bud is known to be invested with the δ form of ENaC at the expense of the α form [36]. Panel E shows immunoreactivity to antibody against ASIC1a, with the Nomarski optics of the same section displayed in Panel D. The taste bud area of the section in Panel E is magnified to a larger black and white image showing the specific labeling of the characteristic spindle-shaped cells within a taste bud. Panel F shows immunoreactivity to antibody against ASIC1β in the taste bud (outlined) and in the plexus, overlaid on a photo of the same section under Nomarski optics. The distribution of the channels PKD2Ll and PKD1L3 in the human fungiform papilla is shown in Panels G, H and I. Panels G and H display immunoreactivity toward the ion channel PKD2L1. A DAPI overlay marks each cell. The arrow in Panel G points to a taste bud. This taste bud appears in Panel H at an approximately five-fold magnification. Note the labeling of the membranes of several taste cells. Panel I shows distribution of immunoreactivity to an antibody made against PKD1L3. It shows the upper portion of a human fungiform papilla using Nomarski optics overlaid with the immunohistochemical label from an antibody against PKD1L3 and DAPI stain. A taste bud is outlined. The label for PKD1L3 appears between cells and is likely not labeling the membrane of the taste bud cells because the label does not appear in the cells, as it would were the antibody recognizing an antigen on the taste cell. The bars on each Panel show the magnification: A & B: the box width is 40 µm, blow-up in Panel B is a 2-fold magnification of the left smaller box; C: 20 µm; D & E: 25 µm with Insert E: 10 µm; F: 25 µm; G: 80 µm; H: 15 µm; I: 15 µm.

Figure 5 shows labeling of human fungiform taste buds by antibodies to PLC-β2, δ-ENaC, ASIC1a, ASIC1β, PKD2L1 and PKD1L3.

The antibodies developed against PLC-β2 labeled only a few cells, but those, very intensely (Panels A and B). Panel A shows the taste bud containing tissue slice in Nomarski optics, while panel B shows the same section under fluorescence. The area surrounded by the box in panel A is shown under fluorescence at the same size in the left of Panel B, and then shown magnified on the right side of Panel B.

Because transduction of sweet, umami and bitter tastes require PLC-β2, the fact that very few cells within the bud react with an antibody to PLC-β2 implies that very few cells act as receptors for sweet, umami, and bitterness in the human fungiform taste bud.

Panel C displays the distribution of δ-ENaC immunoreactivity in taste buds and represents the Nomarski optics picture of a tissue slice overlaid with the immunofluorescence for δ-ENaC. Note that the antibody to the δ-subunit of human ENaC preferentially labels the membranes of the taste bud cells. The cells are labeled both apically and basolaterally. This pattern of apparent δ-subunit labeling at both apical and basolateral ends is in contrast to one recently described [39] which found only apical labeling. Several replications of our experiments confirmed our labeling pattern for δ-ENaC.

Panels D, E and F of Figure 5 demonstrate that antibodies to ASIC1a (Panel E) and to ASIC 1β (Panel F, with the taste bud outlined) label both the taste bud and the plexus below the bud. Panel D shows the clear presence of a taste bud under Nomarski optics. Labeling of the taste bud in Panel E is more readily seen with a magnified black and white photomicrograph (expanded section Figure 5E). The lower plexus area contains connective tissue, nerve fibers and blood vessels. The dot-like appearance of structures between cells of the bud may perhaps indicate the presence of nerve fibers. The occurrence of immunoreactivity for the ASICs in the plexus, within some taste bud cells and in spaces between cells, suggests that the ASICs may not only be involved in receptor events, but that they may also respond to regulatory processes, acting perhaps in a paracrine fashion, similar to that reported recently for glucagon-like peptide [40].

Panels G, H and I show the immunoreactivity to antibodies made against the channels PKD2L1 and PKD1L3 within the human fungiform papilla. Panel G shows apparent distribution of PKD2L1 antibody labeling, with DAPI overlay to show the presence of cell nuclei. The single taste bud, labeled by the antibody to PKD2L1, appears at the arrow. This labeling is more clearly seen in Panel H, where the bud is outlined and where the membrane of several characteristic spindle shaped taste bud cells within that outline are labeled. Panel I shows a Nomarski image overlaid with an antibody against PKD1L3, and overlaid on that the DAPI of the same section. The taste bud is outlined in the center of the section, distinguished as an onion-shaped gathering of elongated cells. While the cells show no labeling, positive label is seen between cells and as rows of dots, often indicative of neural tissues. Although difficult to say at this level of analysis, the label for PKD1L3 is likely not recognizing an antigen on the taste bud cell membrane because the entire cell membrane is not labeled. Rather, it appears as though the antibody is labeling a tissue winding through the taste bud, most likely nerve fibers.

Our RT-PCR results, wherein evidence of transcripts for both PKD2L1 and PKD1L3 was detected in human fungiform papillae of normal subjects (Figure 2), are consistent with our immunohistochemical results. The latter data show only PKD2L1 in taste bud cells (Panel H) and PKD1L3 in areas of the fungiform papillae other than the taste bud cells (Panel I). This suggests that PKD1L3 may be involved in a paracrine fashion in sour sensing, which would explain its undetectability in the sour-ageusic patients. The detection of PKD1L3 in human fungiform papillae (sour normal) is in contrast to results from rodents showing no expression of PKD1L3 in anterior tongue [16]–[18]. On the other hand, rodents do express PKD1L3 in the posterior tongue.

The combined observations from both sour-ageusics, whose fungiform papillae cDNA lacked transcripts for the ASICs and PKDs, and from sour-normals, whose fungiform papillae cDNA clearly expressed transcripts for the ASICs and PKDs, should be of compelling importance in deciphering the sour taste transduction mechanisms in humans.

### Speculations on the sour taste receptor mechanism in human fungiform papillae

The four major observations of this study are as follows: (1) Two patients were documented as completely ageusic to a sour (citric acid) stimulus, yet both showed normal taste responses to sucrose and quinine, while one showed a normal response to NaCl, with the other showing a slightly elevated threshold for the saltiness of NaCl; (2) using the cDNA of fungiform taste papillae from the sour-ageusic patients we failed to detect transcripts for five members of the ASIC family and two PKDs previously associated with sour taste, while, in contrast, (3) using the cDNA of fungiform taste papillae from control individuals able to taste sour stimuli we detected transcripts for the same five ASICs and two PKDs; and finally (4) using cDNA from both the normals and the ageusics, we detected transcripts for β-actin, and for taste bud-related transcripts of PLC-β2, δ-ENaC and the bitter receptor, T2R14.

Taking into account the new data presented here and those in the literature, we can now list and integrate information about sour taste transduction and speculate on the nature of the sour taste receptor.

- The mechanisms for sour taste are likely to be species specific. The literature contains studies showing that not every animal shares the same proton-stimulated oral ion channels, assuming these particular channels to be sour receptors [6].
- The mechanisms for sour taste may also be specific to a particular region of the tongue. Even though in our studies here we found two patients who were totally sour-ageusic, other reports in the literature lead us to speculate that the mechanisms used to detect sourness in anterior and posterior tongue are different, albeit similar [41]. Strengthening this suggestion is the observation we reported on in abstract [24], but will soon report on more fully, that some individuals lack the ability to recognize sourness on the tip of the tongue, but can readily identify the modality using a whole mouth sip-and-spit procedure. There is also the demonstration of salty-sour confusion in the posterior tongue but not in the anterior [42]. Other workers have shown that cells in the taste buds of mouse vallate papillae that responded to NaCl were a subset of sour-responding cells [43]. These observations are consistent with the documented salty/sour confusion.
- That there are specific cell lineages for taste modalities is supported by the very existence of specific ageusias, such as those reported here, and elsewhere in the literature [35]. Recent work in mice, using the technique of cell lineage analysis, points to the same conclusion and strongly suggests that at least some of the different cell types in a taste bud represent distinct lineages of cells, rather than simply being different developmental phenotypes [44].
- Several ion channels have been suggested as sour taste receptors in the literature, in particular the ASICs [8], the PKDs [18], K^+^-channels [45] and Ca^2+^-channels [6]. In most cases these are both region– and species–specific.
- The fact that we could not detect transcripts for every ASIC and PKD probed for in the cDNA from fungiform papillae of the sour-ageusics, yet readily detected these in the cDNA from fungiform papillae of sour-normals, argues for each of these channels playing some role in sour taste. They may not all act as direct receptors for sour taste, yet in some way they may exert their activity subsequent to the receptor step, perhaps in a paracrine fashion, from cells or neurons both at the periphery of the bud and in neurons coursing through the bud. Nevertheless, no matter where they are expressed, the undetectability of transcripts for any of the ASICs or the PKDs in the sour-ageusics suggests that their expression is under the control of a progenitor specific for sour cells.
- Based on these results, we suggest that most or perhaps all of these channels (and possibly others) are necessary for forming sour taste receptors as homomeric and heteromeric complexes. Further, each complex may cover a portion of the dynamic range for sour stimulation, such range being from pH 1.5 to pH 5.5 (much as one would design a buffer system for covering a multi–order-of magnitude range). Strengthening this suggestion is the observation that the pH sensitivity range of the heteromeric ASIC complexes is dependent on their composition [46]. We further speculate that the channels overlap in sensitivity and that some are composed of homomeric and heteromeric complexes of the ASICs and PKD channels. Such complexity may be required to account for the well-documented observation that the human perception of sourness is influenced by all three components of an acid molecule – the proton, the anion *and* the undissociated acid [5], [7]. Precedent for this ensemble model can be found in the multiple factors model of acid signaling in central chemosensitive neurons where multiple ion channels are targets of multiple acidic stimuli [22]. A similar heterogeneous ion channel array is postulated to be responsible for detecting and reacting to the wide pH range encountered in the gastrointestinal tract [23]. Finally there is the example of the classic array of temperature-sensitive TRP channels that allows detection of skin temperature ranging from noxious cold to burning heat [47].
- There is another hint of this underlying complexity in the observation that citric acid at low concentrations (<1 mM) is actually preferred over water by certain strains of mice [48], raising the possibility that there are both “good” and “bad” sour sensations, mediated by different neural mechanisms [17], perhaps in humans as well.

By using the information gleaned from the sour-ageusics, we recognize that we are drawing conclusions based on the inability to detect certain transcripts – i.e. “negative data.” We understand the limitations this puts on our conclusions, yet we also appreciate the opportunity that these sour-ageusics present. These were two patients with very specific sour ageusia. Other taste modalities in these patients were not affected. Such a specific loss may be reflected in the loss or attenuation of modality-specific proteins. We tried, without success, to detect transcripts for some of these putative proteins in the cDNA of taste papillae from these patients. By contrast, we were readily able to detect the expression of these same transcripts in the cDNA of subjects who can taste sour stimuli. Such clear differences in transcript expression lead us to conclude that the proteins corresponding to these transcripts are involved at some point in the transduction pathways resulting in the perception of sourness.

Studies with ASIC2a-null mice have shown that they do not have significant impairment of responses to sour stimuli [49], and this is also the case with PKD1L3-null mice [50]. These findings are consistent with our hypothesis that there are multiple ion channels and regulatory factors involved in sour taste transduction, because a corollary of this hypothesis is that knocking out the gene for any one putative sour taste receptor will have only a limited impact on the neural and behavioral responses to sour stimuli. Indeed, one might have to knock out three or four, or even more, genes simultaneously in order to see an effect. This is not technically feasible at the moment, even in mice. By contrast, this situation is a reality in the case of our two sour-ageusic patients. It is true that these patients are not “seven-gene knockouts” but rather “seven-transcript knockouts” but in the context of taste transduction the net effect is the same – i.e. a profound loss in the ability to taste sour stimuli. Of course, we cannot tell at this time if all the seven genes need to be knocked out for the sour ageusia to manifest itself. It is possible that knocking out a smaller number may be sufficient to produce the sour ageusia. The remaining genes may be unexpressed because, with the lack of sour receptors, their role in taste transduction is no longer important. It is also likely that the loss of these genes is a consequence of the primary loss of sour-sensing cells. Future studies with other sour-ageusic individuals will be required to determine which taste cell type is affected. Efforts should now be made to identify more sour-ageusic individuals in the general population and repeat the psychophysical and molecular experiments we have described in this report.

## Materials and Methods

### Subjects

Five individuals participated in this study. Two (Patients 8689 and 8716) were seen in the Monell-Jefferson Taste & Smell Clinic (MJTSC), while three others (Subjects 45, 65 and 49) were healthy volunteers acting as controls. All five individuals gave their informed consent to both psychophysical testing and the tongue biopsy. Patient 8689 (deceased) was an African American male, age 83 years. Patient 8716 was a white female, age 62. This patient has given written informed consent (as outlined in the PLoS consent form) to publication of her case details. Subject 45 was a white male, age 62. Subjects 65 and 49 were Asian males, ages 44 and 58 years respectively. All three control subjects have also given written consent (as outlined in the PLoS consent form) to publication of their case details.

The control subjects verified that they could correctly identify 5 mM and 15 mM citric acid as sour both with the tongue tip alone and by whole mouth sip-and-spit. To perform the tongue-tip procedure, three small and identical Petri dishes were set before the subjects, one containing 10 ml water, another containing 10 ml of 5 mM citric acid, and the third containing 10 ml of 15 mM citric acid. Subjects were asked to touch their tongue to the bottom of each dish and identify those dishes containing a sour stimulus, and then identify the most potent sour stimulus.

### Psychophysical studies

The two patients seen at the MJTSC received a complete medical work-up. They were given the Clinic's standard screening sensory tests as a first pass procedure designed to detect likely problems of both taste and smell. Both were subsequently given a more rigorous threshold detection evaluation.

The screening test for taste, involving direct scaling of perceived intensity of suprathreshold concentrations of four taste stimuli, has been described previously [51]. In brief, patients sampled 10 ml of three concentrations of a sour stimulus (citric acid at 1.8, 5.6 and 18.0 mM), three concentrations of a salty stimulus (NaCl at 100, 320 and 1000 mM), three concentrations of a sweet stimulus (sucrose at 100, 320, and 1000 mM), and three concentrations of a bitter stimulus (quinine sulfate at 0.008, 0.056, and 0.18 mM). Stimuli of different qualities were presented to determine whether abnormal taste perception was particular to one or more taste modalities rather than a general loss of taste. Patients sampled all 12 stimuli (in random order with a water rinse between each stimulus) using a whole-mouth sip-and-spit procedure. In all cases, patients selected the term that best described the taste quality of each sample from the following list: sweet, sour, salty, bitter or no taste (forced choice). This evaluation of the 12 stimuli was then repeated using another random order. Detection thresholds for the four stimuli named above were obtained using a forced – choice, staircase procedure. Stimulus concentrations were set so that successive solutions differed by 0.25 log units. Concentrations ranged from 1.0×10^−5^ M to 1.0 M for sucrose and NaCl; from 1.0×10^−6^ M to 0.018 M for citric acid; and 5.6×10^−9^ M to 1.8×10^−4^ M for quinine sulfate. On each trial, a blank (filtered, deionized water) and a cup containing a taste stimulus were presented, with subjects being required to identify the cup which they believed to contain the stimulus. A single incorrect response caused an increase in concentration on the next trial while two correct responses caused a decrease. Five such reversals designated the end of the procedure. Thresholds were determined as the mean of the dilution step values of the last four reversals. These psychophysical procedures were approved by the Institutional Review Board of Thomas Jefferson University, Philadelphia, PA.

### Biopsy of human fungiform papillae

The general procedure has been previously described [52]. In brief, subjects first read and understood a consent form, signing it in the presence of the Principal Investigator. The biopsy itself was performed by an oral surgeon. While seated, the subject received an injection of 0.25 ml lidocaine sub-dermally into the anterior one-third of the tongue. The site of injection was at a position distal from the collection site to avoid possible interference of lidocaine with subsequent experiments. Within 2–3 minutes after injection, a small area (∼1 sq. cm.) of the tongue surface became numb to a blunt probe. Using small spring scissors (Roboz) the surgeon clipped out the top half of 6–8 fungiform papillae from each subject, and these were immediately placed in a tube containing 1 ml RNAlater™ (Ambion) and stored at 4°C for no more than 72 hours. The same biopsy procedure was used to collect human fungiform papillae for immunohistochemistry in sour normal subjects. Subjects reported no untoward after-effects of the biopsy, and no noticeable alteration in taste perception. The biopsy procedure and overall protocol were approved by Schulman Associates Institutional Review Board, Cincinnati, OH, and by the Institutional Review Board of Thomas Jefferson University, Philadelphia, PA.

### Extraction of RNA from human fungiform papillae

The excised papillae from each subject were removed from the solution of RNAlater ™ and homogenized in an all-glass tissue grinder containing 1 ml Trizol™ reagent (Invitrogen). Total RNA was extracted according to the manufacturer's instructions and the RNA pellet was resuspended in water. It was treated with DNase to remove genomic DNA contamination, using the Turbo DNA-free ™ kit (Ambion). The RNA preparation was used as the substrate for first strand cDNA synthesis using the reverse transcriptase Superscript III ™ (Invitrogen). An aliquot of RNA lacking reverse transcriptase was simultaneously carried through the protocol. The cDNA samples thus obtained were used as templates for amplification.

### Amplification of specific transcripts from human fungiform papillae

Primers used in this study are shown in Table 1. Amplification was performed with the eLONGase™ Amplification System (Invitrogen) in accordance with the manufacturer's instructions. Cycling parameters using the Perkin-Elmer PE-480 were as follows: initial denaturation at 94°C for 30 seconds; followed by 50 cycles of: denaturation at 94°C for 30 seconds; annealing at 53–62°C (depending on the primer pair) for 30 seconds; and extension at 68°C for 30 seconds to 2 minutes (depending on the expected size of the transcript).Where reaction products from agarose gel electrophoresis were of the predicted size, the band was excised and DNA was extracted using the Qiaquick™ Gel Extraction kit (Qiagen). The purified amplification product was subcloned into pGEM-T-Easy™ (Promega) and transformed into *E.coli* cells of the JM-109 strain. Plasmid minipreps were performed with the QuantumPrep™ kit (BioRad). Restriction analysis was carried out to identify those clones carrying inserts. For all the transcripts probed in this study, 3–6 independent clones were analyzed. Plasmids were sequenced at the University of Pennsylvania DNA Sequencing Center. Bioinformatic analysis of the sequences was performed using BLAST and other freeware.

### Analysis of genomic DNA

Genomic DNA was extracted from buccal scrapings using the BuccalAMP™ kit (Epicentre), in accordance with the manufacturer's instructions, and aliquots of the extracts were subsequently used for amplification experiments.

### Immunohistochemistry

Biopsies of fungiform papillae from the anterior human tongue of sour-normal individuals were excised and fixed in 4% paraformaldehyde in phosphate-buffered saline (PBS) for 1–2 hours, then cryoprotected in a sucrose series. The biopsies were cut in 10 µm sections and placed onto Starfrost Adhesive slides (Mercedes Medical) and stored at −30°C. To ascertain whether a taste bud would likely be on any given section, the section immediately adjacent to the one of interest was stained for ATPase [53]. Once a given section was shown to possess a taste bud, the slides were removed from −30°C and dried at 40°C for 20 min. They were washed for 10 minutes in 10 mM PBS, pH 7.4. To block nonspecific binding, sections were incubated at room temperature with SuperBlock blocking buffer (Pierce, catalog #37517) for 4 hours. Sections were incubated with primary antibody diluted in 10% SuperBlock overnight at 4°C in a humidified chamber, followed by secondary antibody conjugated to a fluorescence probe (Cy3 goat anti-rabbit IgG, Jackson Immuno Research Lab or Alexa Fluor 633 goat anti-rabbit IgG, Molecular Probes Lab) in 1% SuperBlock for 1 hour at room temperature. The sections were washed twice with PBS followed by 1–2 rinses with MilliQ water, then mounted with Vectorshield or Vectorshield with DAPI (Vector Laboratories, Burlingame, CA). Controls lacking the primary antibody were included in the protocol. Antibodies used (from Santa Cruz Inc., Lifespan Biosciences Inc. and Abcam Inc.) included anti-Acid-Sensing Ion Channel (ASIC1a and ASIC1β), anti-Epithelial Sodium Channel (δ-ENaC), anti-PLC-β2, anti-PKD1L3 and anti-PKD2L1. Images were taken using a Leica TCS SP2 Spectral Confocal Microscope (Leica Microsystems Inc., Mannheim, Germany).
